# A New Method for Land Vehicle Gravimetry Using SINS/VEL

**DOI:** 10.3390/s17040766

**Published:** 2017-04-04

**Authors:** Ruihang Yu, Meiping Wu, Kaidong Zhang, Shaokun Cai, Juliang Cao, Minghao Wang, Lin Wang

**Affiliations:** College of Mechatronics and Automation, National University of Defense Technology, Changsha 410073, China; yuruihang@nudt.edu.cn (R.Y.); zhangkaidong@nudt.edu.cn (K.Z.); shaokuncai@nudt.edu.cn (S.C.); jlcao@nudt.edu.cn (J.C.); wang900304@nudt.edu.cn (M.W.); wanglin11@nudt.edu.cn (L.W.)

**Keywords:** gravimetry, SINS, SGA-WZ02, velometer, internal and external accuracy

## Abstract

The use of Global Navigation Satellite System (GNSS) data for land vehicle gravimetry tests is challenged by complicated environments. A new approach for land vehicle gravimetry using a Strapdown Inertial Navigation System and velometer-integrated navigation computation (SINS/VEL) without using GNSS information has been put forward. Aided by the velometer with continuous longitudinal velocity output instead of GNSS signals, a SGA-WZ02 strapdown gravimeter that used the SINS/VEL method was tested in 2015. Four repeated lines were measured along a south-north direction highway in Eastern Changsha to verify the new method’s feasibility and performance. The gravity disturbance results showed an internal accuracy in scalar gravimetry about 1.17 mGal and 1.91 mGal for external accuracy assessment, with a spatial resolution of 1.7 km. Comparing this new method with the traditional SINS/GNSS gravimetry approach, it appeared that the results using SINS/VEL showed comparable internal and external accuracy. Theoretical analysis and practical test results showed that the new method was feasible for gravity determination by land dynamic vehicle.

## 1. Introduction

Nowadays, gravity data are needed for applications such as geophysical and geodesy studies. Continuous gravimetric data is quite necessary in geodesy for geoid determination and gravity field model refinement. Moving base gravimetry is playing an important role in geo-information surveys [1,2,3,4,5,6]. In solid Earth geophysics, the major requirement for spatial resolution is between 10 and 100 km, but in oil exploration, the detection of local gravity anomalies with typical extensions of 1 to 10 km is of specific interest [7]. Over the years airborne gravimetry has been widely studied with the rapid development of Strapdown Inertial Navigation System and Global Navigation Satellite System (SINS/GNSS). Several representative gravimeters with different principles such as AIRGrav, GT-1A, LCR, SISG and SGA-WZ gravimeters have been applied for gravity surveys and geological exploration all over the world [8,9,10,11,12,13,14]. 

Compared with satellite and airborne gravimetry, cars used for land vehicle gravimetry are more dynamic than satellites and aircrafts. With the restrictions of the roads, cars cannot be used everywhere especially in inaccessible areas, however, land vehicle gravimetry has an advantage in altitude. Theoretically, the gravity signal becomes stronger when the gravimeter is closer to the Earth’s surface. Besides, ground vehicle gravity determination costs much less time and funds. Given the car’s lower speed and altitude, land vehicle gravimetry provides an effective option and shows rosy prospects for obtaining high resolution gravity data in local regions. A land-based SINS/GNSS Gravimetry test was carried out in Taiwan and validated that the land-based gravimetry has potential for groundwater resource detection [15]. In 2005, Li et al. carried out several land vehicle tests in West Montana and got inspiring results that were at the level of 1 mGal repeatability accuracy and about 2–3 mGal respecting to the control data [16]. To the authors’ knowledge, this was the first time that a gravimetric system worked in a ground vehicle. However, implementing terrestrial gravity surveys has to face more complicated conditions than airborne gravimetry, especially in the aspect of GNSS observation environments [17]. Besides, terrestrial gravimetry suffers by high frequencies features around the gravimeter and the results are sensitive to local gravitational features. In some special applications, GNSS even cannot be applied to gravimetry experiments for some reasons, such as in tunnels or underwater where cannot receive the GNSS signal or even GNSS is not permitted to use during the test. On the other hand, disadvantages of GNSS weak dynamic responses and easily be blocked or cheated will severely lead to decrease the gravimetry accuracy. If one could find a method that makes gravimetry not totally depend on GNSS restrictions, it would greatly expand the application range of land vehicle gravimetry. Considering applying gravimeters for multiple environments or even no-GNSS signal situations, our purpose in this paper was to find a new method for land vehicle gravimetry without using GNSS receivers.

In traditional airborne and vehicle gravimetry, GNSS plays an important role to provide high precision navigation parameters such as kinematic position, velocity, acceleration, etc. Actually in last few years, benefiting from the rapid development of GNSS technology, airborne gravimetry all over the world has maintained rapid growth. It is not easy to find an alternative way not to use GNSS for aiding SINS in certain special applications. Unlike aircraft, cars have the characteristic of non-holonomic constraints in the course of motion, which makes car’s motion different from that of aircraft. In the applications of SINS, the inherent disadvantage that the positioning error accumulates with time lapsing still exists. On the premise of not using GNSS, methods of INS/Odometer (INS/OD) integrated navigation computation for compensating errors have been put forward for vehicle navigation in recent years [18,19]. Most of these researches focused on limiting error divergence and improving navigation precision. In this paper, velometer which is similar to odometer in principle has been introduced to aid the strapdown gravimeter SGA-WZ02, and a method of Strapdown Inertial Navigation System and Velometer integrated computation (SINS/VEL) for land vehicle gravimetry has been presented.

It should be pointed out that, this method is a continuation of the work published by Yu et al. [20]. In [20], GNSS results were specially analyzed and results which were comparable at the level of 1–3 mGal for internal and external accuracy showed that this gravimeter can be applied for land vehicle gravimetry by using traditional SINS/GNSS method. Differences and improvements in this paper are aiming at applying this strapdown gravimeter for no-GNSS-environment applications, the authors dug deep and presented a new method for land vehicle gravimetry.

The paper is organized as follows: the principle of land vehicle gravimetry using SINS/VEL is briefly introduced in Section 2. Experiment including gravimeter description and test details are shown in Section 3. Preliminary results and comparisons of different methods are discussed in Section 4. Conclusions are made in Section 5. It is necessary to develop different methods for dealing with different conditions in land vehicle gravimetry. This new method using SINS/VEL provides a promising way to implement local geo-information surveys using land vehicles.

## 2. Principle

Choosing north-east-down coordinate system as the navigation frame (n-frame), the model of moving base gravimetry is expressed by Equation (1):

(1) $$\delta g^{n}={\dot{v}}^{n}-C_{b}^{n}f^{b}+(2\omega_{ie}^{n}+\omega_{en}^{n})\times v^{n}-\gamma^{n}$$

where $v^{n}$ and ${\dot{v}}^{n}$ are the velocity and acceleration of vehicle in n-frame, $f^{b}$ is the specific force sensed by accelerometers in body frame (b-frame), $C_{b}^{n}$ is the transformation matrix which rotates $f^{b}$ from b-frame to n-frame, $\omega_{ie}^{n}$ is angular velocity of the earth respecting to the n-frame and $\omega_{en}^{n}$ is rotation rate of the n-frame due to vehicle rate over the ellipsoid, introducing γ as the normal gravity vector, then $\delta g^{n}$ is the gravity disturbance vector in n-frame [20,21].

In this paper, only scalar gravimetry will be discussed in detail. Expanding Equation (1) to three directions in n-frame, gravity disturbance in down direction $\delta g_{D}$ can be written as:

(2) $$\delta g_{D}={\dot{v}}_{D}-f_{D}+(2\omega_{ie}\cdot \cos L+\frac{v_{E}}{R_{N}+h})\cdot v_{E}+\frac{v_{N}^{2}}{R_{M}+h}-\gamma_{D}$$

where $f_{D}$ is the down direction of specific force, $v_{N}$, $v_{E}$ and $v_{D}$ are the north, east and down velocity in three directions, L and h are latitude and height in geocentric coordinate system, $R_{M}$ and $R_{N}$ are the meridian and prime vertical radius of curvature, $\gamma_{D}$ is the normal gravity and the earth rotation rate is represented by $\omega_{ie}$.

Generally, $v^{n}$, ${\dot{v}}^{n}$, $\omega_{ie}^{n}$, $\omega_{en}^{n}$, $\gamma^{n}$ in Equation (1) can be calculated by GNSS positioning, but considering no-GNSS situation in land vehicle gravimetry, these variables should be calculated by another aiding sensor. Velometer sensor which is mounted at the vehicle can provide longitudinal velocity $v_{d}$ information. For this purpose, velometer is introduced in this paper. According to the non-holonomic constraints in the course of vehicle motion, the transverse velocity and the vertical velocity are both zero which means the velocity in vehicle front-right-down frame (m-frame) can be expressed as $v_{velo}^{m}={\left[\begin{array}{ccc} v_{d} & 0 & 0 \end{array}\right]}^{T}$. Then the vehicle’s velocity in n-frame $v_{velo}^{n}$ can be expressed by Equation (3):

(3) $$v_{velo}^{n}=C_{b}^{n}C_{m}^{b}v_{velo}^{m}$$

where the transformation matrix $C_{m}^{b}$ rotates the vehicle velocity vector $v_{velo}^{m}$ from m-frame to b-frame. Setting the true transformation matrix is $C_{b}^{n}$, while ${\tilde{C}}_{b}^{n}$ is the estimated matrix. Setting $\varphi^{n}={\left[\begin{array}{ccc} \delta \alpha & \delta \beta & \delta \gamma \end{array}\right]}^{T}$, then $\left(\varphi^{n}\times \right)$ is the skew symmetric matrix which represents the attitude errors of transformation matrix $C_{b}^{n}$. Expression of $\left(\varphi^{n}\times \right)$ is shown as Equation (4):

(4) $$\left(\varphi^{n}\times \right)=\left[\begin{array}{ccc} 0 & -\delta \gamma & \delta \beta \\ \delta \gamma & 0 & -\delta \alpha \\ -\delta \beta & \delta \alpha & 0 \end{array}\right]$$

Assuming the installation Euler roll-pitch-yaw angle vector between velometer m-frame and b-frame is ${\left[\begin{array}{ccc} \eta & \theta & \psi \end{array}\right]}^{T}$, then the installation Euler error angle of $C_{m}^{b}$ is $\sigma ={\left[\begin{array}{ccc} \delta \eta & \delta \theta & \delta \psi \end{array}\right]}^{T}$. Making some reasonable approximations such as sinδθ≈δθ cosδθ≈1 (because δθ is a small angular error), the equation of vehicle’s velocity in n-frame ${\tilde{v}}_{velo}^{n}$ can be written and simplified as follows [22]:

(5) $$\begin{array}{l} {\tilde{v}}_{velo}^{n}={\tilde{C}}_{b}^{n}{\tilde{C}}_{m}^{b}{\tilde{v}}_{velo}^{m}=\left[I-\left(\varphi^{n}\times \right)\right]C_{b}^{n}\left[I-\left(\sigma \times \right)\right]C_{m}^{b}\left(1+\delta k_{velo}\right)v_{velo}^{m}\\ \;=\left[I-\left(\varphi^{n}\times \right)\right]C_{b}^{n}\left[I-\left(\sigma \times \right)\right]C_{m}^{b}\left(1+\delta k_{velo}\right)v_{velo}^{m}\\ \;=\left[I-\left(\varphi^{n}\times \right)\right]C_{b}^{n}\left[\begin{array}{c} \cos\tilde{\theta }\cos\tilde{\psi }\\ \cos\tilde{\theta }\sin\tilde{\psi }\\ -\sin\tilde{\theta } \end{array}\right]\left(1+\delta k_{velo}\right)v_{d}\\ \;=\left[I-\left(\varphi^{n}\times \right)\right]C_{b}^{n}\left[\begin{array}{c} \cos(\theta +\delta \theta )\cos(\psi +\delta \psi )\\ \cos(\theta +\delta \theta )\sin(\psi +\delta \psi )\\ -\sin(\theta +\delta \theta ) \end{array}\right]\left(1+\delta k_{velo}\right)v_{d}\\ \;=\left[I-\left(\varphi^{n}\times \right)\right]C_{b}^{n}\left[\begin{array}{c} \cos\theta \cos\psi -\sin\theta \cos\psi \cdot \delta \theta -\cos\theta \sin\psi \cdot \delta \psi \\ \cos\theta \sin\psi -\sin\theta \sin\psi \cdot \delta \theta +\cos\theta \cos\psi \cdot \delta \psi \\ -\sin\theta -\cos\theta \cdot \delta \theta \end{array}\right]\left(1+\delta k_{velo}\right)v_{d}\\ \;=v_{velo}^{n}-\left(\varphi^{n}\times \right)v_{velo}^{n}+v_{d}C_{b}^{n}\left[\begin{array}{cc} -\cos\psi \sin\theta & -\cos\theta \sin\psi \\ -\sin\theta \sin\psi & \cos\theta \cos\psi \\ -\cos\theta & 0 \end{array}\right]\left[\begin{array}{c} \delta \theta \\ \delta \psi \end{array}\right]+\delta k_{velo}v_{velo}^{n} \end{array}$$

Then the error equation of $\delta v_{velo}^{n}$ is simplified as:

(6) $$\begin{array}{ll} \delta v_{velo}^{n} & ={\tilde{v}}_{velo}^{n}-v_{velo}^{n}\\ & =-\left(\varphi^{n}\times \right)v_{velo}^{n}+v_{d}C_{b}^{n}\left[\begin{array}{cc} -\cos\psi \sin\theta & -\cos\theta \sin\psi \\ -\sin\theta \sin\psi & \cos\theta \cos\psi \\ -\cos\theta & 0 \end{array}\right]\left[\begin{array}{c} \delta \theta \\ \delta \psi \end{array}\right]+\delta k_{velo}v_{velo}^{n}\\ & =v_{velo}^{n}\times \varphi^{n}+v_{d}C_{b}^{n}M_{\sigma }\delta \sigma +\delta k_{velo}v_{velo}^{n} \end{array}$$

where:

(7) $$M_{\sigma }=\left[\begin{array}{cc} -\cos\psi \sin\theta & -\cos\theta \sin\psi \\ -\sin\theta \sin\psi & \cos\theta \cos\psi \\ -\cos\theta & 0 \end{array}\right],\delta \sigma =\left[\begin{array}{c} \delta \theta \\ \delta \psi \end{array}\right]$$

Analyzing Equation (6), we can find that $\delta v_{velo}^{n}$ has relations to the velocity $v_{velo}^{n}$, error vector φ, Euler pitch angle θ, yaw angle ψ and scale factor error $\delta k_{velo}$, but it has nothing to do with Euler roll angle η. In general, the accuracy of velocity error $\delta v_{velo}^{n}\leq 0.03m/s$ is required for airborne gravimetry according to reference [3]. Considering lower velocity of land vehicle (about 10 m/s) than that of aircraft (about 60 m/s), the velocity error requirement can be properly loosened. In land vehicle gravimetry test, if we still intend to achieve the requirement $\delta v_{velo}^{n}=0.03m/s$, allowable errors in Equation (6) are listed as follows.

With the rapid development of studying high-precision inertial sensors such as accelerometers and gyroscopes, it appears that the requirements of attitude error in Table 1 could be achieved [23]. In recent years, many researchers have also focused on calibrating the odometer parameters and installation angles [24,25]. Calibrating the installation matrix $C_{m}^{b}$ offline, $\delta \theta \leq 1\times 10^{-3}$, $\delta \psi \leq 1\times 10^{-3}$ and $\delta k_{velo}\leq 1\times 10^{-3}$ can be also satisfied for the accuracy requirements of velocity, so it is feasible to achieve the measuring accuracy requirements for land vehicle gravimetry.

After obtaining the velocity in n-frame $v_{velo}^{n}$, preliminary acceleration of the vehicle ${\dot{v}}_{velo}^{n}$ can be calculated by first-order difference, and other variables mentioned in Equations (1) and (2) which are functions of positions and velocities can also be calculated. Implementing SINS/VEL integrated navigation computation by Kalman filtering estimation, and then gravity disturbance $\delta g_{D}$ can be obtained by Equation (2).

## 3. Experiments

A gravimetry experiment was implemented based on the SGA-WZ02 strapdown gravimeter in 2015. The SGA-WZ02 strapdown gravimeter was first developed by National University of Defense Technology (NUDT, Changsha, China) in 2014. Equipped with three pairwise orthogonal quartz flexibility accelerometers and one triad of navigation-grade Ring Laser Gyroscopes (RLG), the Inertial Measurement Unit (IMU) unit outputs data at a rate of 200 Hz for a logging system. Accelerometer stability is at the level of 0.6 mGal/day with the help of a precise thermal control system. The stability of each RLG was ±0.004 °/h and random noise was $\pm 0.002\;{}^{\circ}/\sqrt{h}$. With power supplied by an automotive uninterrupted power supply (UPS) electrical source, it can be ensured that the system can work continuously during the whole test. More details on the SGA-WZ02 system can be found in [20].

In the former research on land vehicle gravimetry based on the SGA-WZ02, GNSS data was used and carefully analyzed. Forgetting about GNSS, our objective in this paper is obtaining the gravity disturbance without GNSS data, so the KISTLER Correvit^®^ S-350 optical velometer is introduced in this test. The S-350 optical sensor is designed for direct, slip-free measurement of longitudinal and transverse vehicle dynamics. Featuring high-quality optical elements and state-of-the art high-performance signal processing based on Digital Signal Processor (DSP) and Field Programmable Gate Array (FPGA), it can produce longitudinal velocity with good accuracy on all standard testing surfaces, even under the most challenging conditions [26]. Receiving time synchronization signal from SGA-WZ02 gravimeter by RS-232 serials communication, the velometer transmits real-time measured velocity information at a rate of 100Hz to data logging system. Working together with strapdown gravimeter, velometer can provide the car’s longitudinal velocity during the test. Typical performance specifications of S-350 sensor are listed in Table 2.

**Figure 1:**
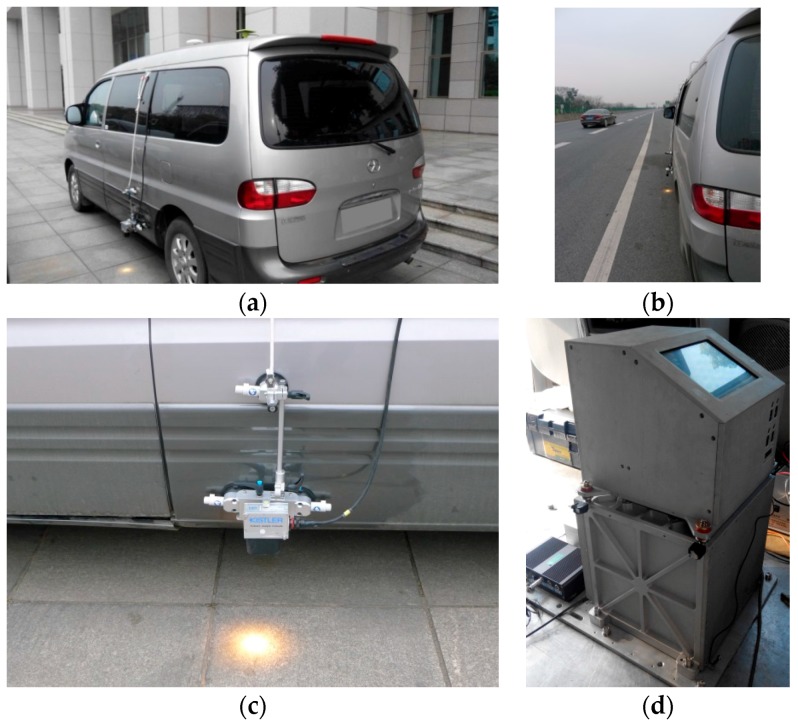
(**a**) Test car equipped with SGA-WZ02 (in the cabin) and KISTLER Correvit^®^ S-350 velometer; (**b**) Test car is driving on the highway; (**c**) The working velometer installed along the side door of the car by versatile mounting frame; (**d**) SGA-WZ02 installed in the cabin of test car.

The test car equipped with the SGA-WZ02 system and Correvit^®^ S-350 velometer is shown in Figure 1. The Correvit^®^ S-350 sensor was installed 350 mm above the road by three suckers which made the velometer securely fixed along the side door of the test car. During the whole test, the velometer sensor was working well and fixed firmly. Calibrating the velometer installation matrix $C_{m}^{b}$ offline, the Euler pitch angle is θ=0.882°, Euler yaw angle is ψ=0.659°, and the scale factor is $k_{velo}=0.998$.

**Figure 2:**
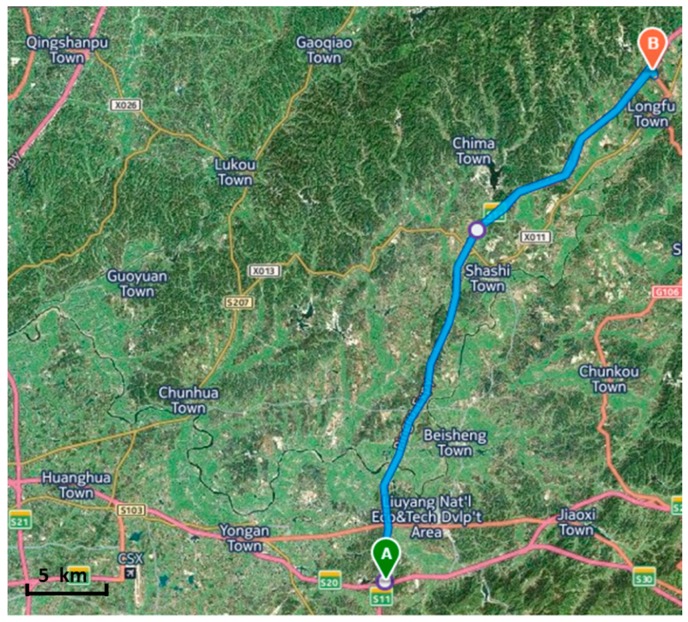
Survey map of the test in Eastern Changsha.

Figure 2 shows trajectories of the test in Changsha, Hunan Province. Driving along a south-north direction highway for two laps, we have measured four repeated profiles. The available distance of each profile was about 35 km. During the whole test, smooth traffic flow helped the car maintain the average speed at about 40 km/h. It is necessary to note that we have built GNSS master/rover stations and also collected the positioning data. Results calculated by GNSS positioning and SINS/GNSS computation were only used for external reference in this paper. 

Besides, the high accuracy gravity reference data along the tested road was established by using a CG-5 gravimeter produced by the Scintrex^TM^ Company (Concord, ON, Canada). This high accuracy gravity data can be repeat used as the reference in the future if further gravimetry test is still carried out along this road. More details about test description and establishing reference data with gravity conjunction can be found in [20].

## 4. Results and Discussion

### 4.1. Data Processing

**Figure 3:**
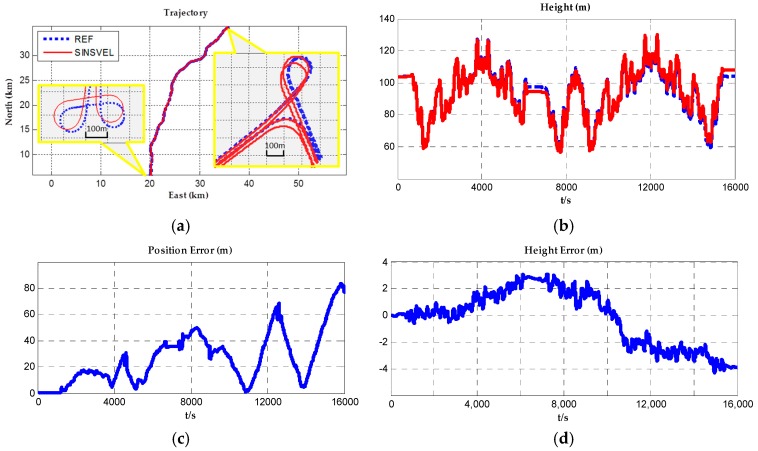
Position and error plots of SINS/VEL computation comparing with the reference data.

Applying SINS/VEL integrated navigation computation, navigation parameters such as position, velocity and acceleration can be obtained. The navigation results including the horizontal position and the height profile were shown in Figure 3a,b. To evaluate the validity of positioning results, taking former GNSS results as the reference, position errors in horizontal and height directions were shown in Figure 3c,d. Comparing with the reference data, the horizontal error ranged from 0 to 80 m and the range of height error was about −4 to 3 m. In Figure 3a, zooming in the typical sections of the trajectory to 100 times (then each grid in yellow boundaries represents 100 m), details show that there are really few mismatches between the results and the reference data. The initial alignment errors and long-term drifts of inertial sensors might be the main reasons that caused the positioning errors. The disadvantage of SINS/VEL in positioning accuracy especially in height errors, limits the land gravimetry test duration to only several hours.

**Figure 4:**
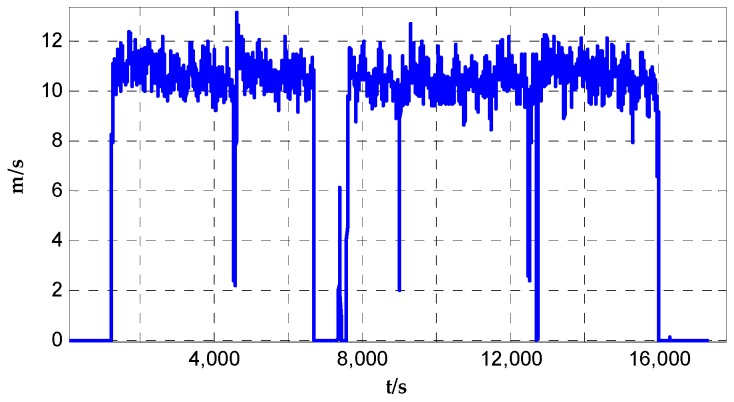
Profile of raw velocity output by S-350 velometer.

The raw velocity profile output by Correvit^®^ S-350 velometer $v_{velo}^{m}$ is shown in Figure 4. The car’s speed ranges 10–12 m/s during the dynamic test duration. Meanwhile, the attitude transformation matrix $C_{b}^{n}$ can be calculated while in the SINS/VEL integrated computing processing, and then the velocity of test car in n-frame $v_{velo}^{n}$ can be obtained.

**Figure 5:**
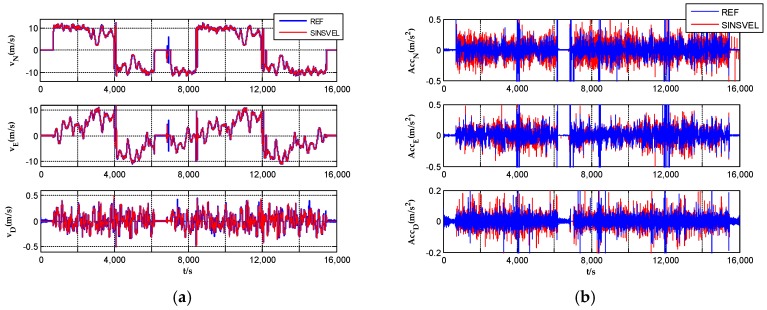
(**a**) Velocity profiles in three directions comparing with the reference in n-frame; (**b**) Acceleration profiles comparing with the reference in n-frame.

Velocity and acceleration profiles in n-frame $v_{velo}^{n}$ and ${\dot{v}}_{velo}^{n}$ are shown in Figure 5. Comparing the calculated velocity and acceleration with reference data obtained by GNSS, all the profiles show quite similar variation tendencies. Noise of velocity calculated by SINS/VEL and GNSS seems to be nearly at the same level, while acceleration obtained by SINS/VEL shows a little larger noise than that from GNSS. To a certain extent, these figures indicate that the new method is efficient and reliable.

**Figure 6:**
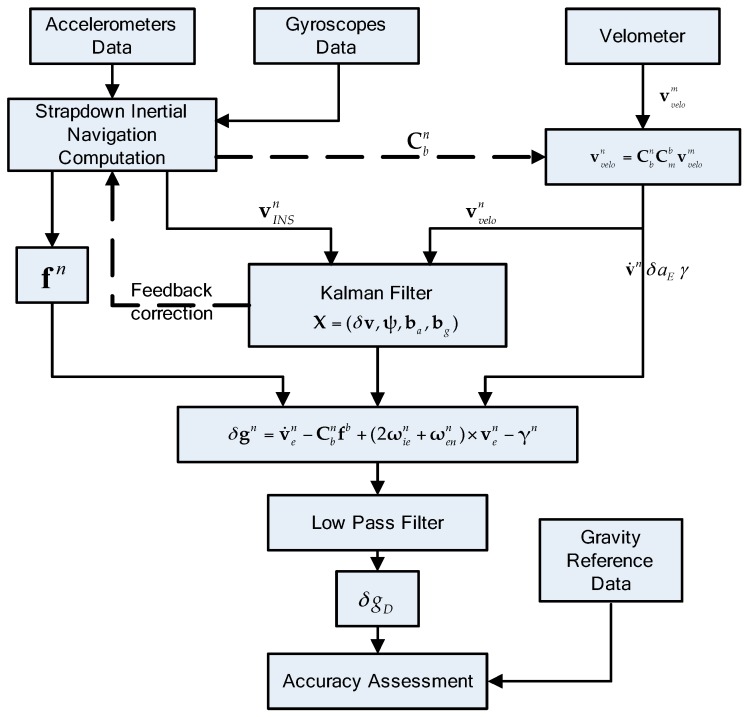
Data processing flow diagram.

Figure 6 shows the whole data processing flow diagram. In this flow diagram, velometer provides the continuous longitudinal velocity $v_{d}$ information. The transformation matrix $C_{b}^{n}$ calculated from strapdown inertial navigation computation is applied for converting the car’s velocity from m-frame to n-frame. Compensating the lever-arm errors between gravimeter and velometer, variables needed in Equation (1) such as velocity $v_{Velo}^{n}$, acceleration ${\dot{v}}^{n}$, eotvos correction $\delta a_{E}$ and normal gravity γ can be calculated. Taking the velocity error between $v_{INS}^{n}$ and $v_{velo}^{n}$ as the measurement vector, Kalman filter was chosen as the estimation algorithm for SINS/VEL integrated navigation. Preliminary gravity disturbance calculated by Equation (1) is mixed up with plenty of high frequency noises and should be filtered by a low-pass Finite Impulse Response (FIR) filter. Since the SGA-WZ02 is basically a relative measurement gravimeter, the bias and other additional long-term uncompensated systematic errors cannot be determined unless an absolute gravity point is available [11]. In order to eliminate these residuals, an endpoint-matching bias correction was implemented with the CG-5 gravimeter gravity conjunction extending the known absolute gravity point to the test area. Taking advantage of the high accuracy external gravity reference which was built previously, gravity disturbance of repeated lines can be calculated for internal and external accuracy assessments.

### 4.2. Test Results and Analysis

**Figure 7:**
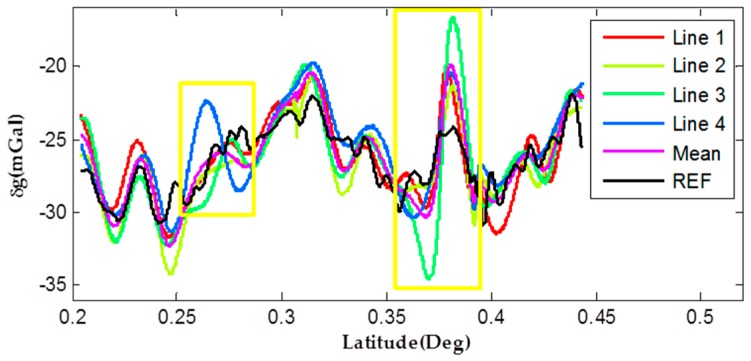
Comparisons of filtered gravity disturbances.

As shown in data processing flow diagram, useful gravity disturbance hides in the raw results. To extract the low-frequency gravity signal, high-frequency noise should be filtered by a low pass FIR filter. Internal and external accuracy can be used for evaluating the performance of this new method. Benefitting from the former reference data establishing work, high precise gravity control data has been obtained that makes it convenient to evaluate the objective accuracy performance. In this test, four repeated lines were evaluated for internal and external accuracy computation. Citing the 300 s FIR filtered results as an example, gravity disturbances are shown in Figure 7. 

In this paper, relative coordinates was chosen to present the X-axis “Latitude”. Details of accuracy assessments are shown in Table 3. The total internal Root Mean Square (RMS) of four repeated lines is 1.17 mGal, and the external RMS is 1.91 mGal. Considering the 300 s FIR filter length and average 40 km/h speed of test car, the gravity spatial resolution is about 1.7 km. Equations on calculating internal and external gravity consistency can be found in [20,27].

Analyzing Figure 7 and Table 3, we can find that the overall trend of the four measured lines is quite similar to the reference data and differences of external consistency are at the level of several mGal. These results indicate that the strapdown gravimeter using SINS/VEL method is feasible and reliable for gravimetry without taking advantage of the GNSS information. However, analyzing Figure 7, we could see that the errors turned large in several regions along the highway such as the blue line on the “Latitude” axis about 0.26 and the green line in the range of 0.36 to 0.38 on latitude axis (see the details within yellow boundaries in Figure 7). These mismatching errors maybe caused by drifting errors of long-time-working inertial sensors or unstable velometer measurement errors because of the changing environments during the test.

**Figure 8:**
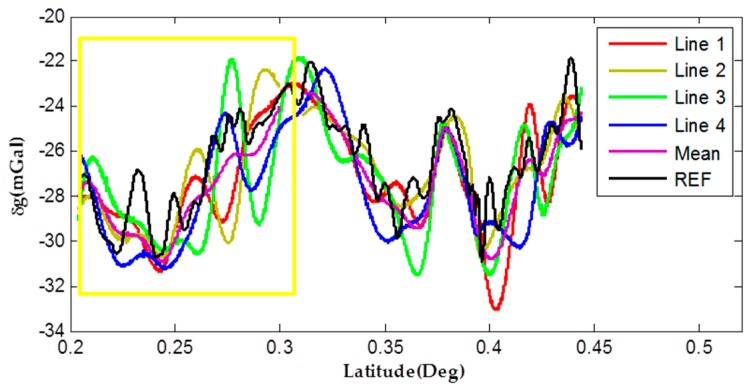
Former gravity disturbances using SINS/GNSS method.

Generally speaking, when a new method is presented, it is necessary to compare the performance with former traditional approach. Former results with the same spatial resolution calculated by SINS/GNSS are shown in Figure 8 [20]. The differences of internal consistency are 1.22 mGal, and meanwhile the external accuracy comparing with reference data is 1.74 mGal.

Comparing Figure 7 with the former profiles in Figure 8, it appears that the new method using SINS/VEL shows comparable internal consistency accuracy. The “Latitude” axis ranging from 0.20 to 0.32 represents the southern part of the tested road where is surrounded by hills (see the details within yellow boundary in Figure 8). Analyzing both results in details, it seems that the new method shows comparable repetitiveness and a little less error than the former results. This is might because unfavorable GNSS observation environment in this hilly area leads to reduced positioning quality, and eventually results in making the final gravity disturbance undulating around the reference data. Results comparisons of this area indicate that the SINS/VEL has an observation advantage over the SINS/GNSS method which is frequently influenced by GNSS observation environment. On the other hand, disadvantages still exist in the new SINS/VEL method, and the result for external accuracy assessment is a little worse. Non-ideal external accuracy indicates that there are still errors hiding deeply in the useful signals. Objectively speaking, GNSS still has the better capability of positioning. Results calculated by SINS/GNSS still show better accuracy than those obtained by SINS/VEL method. Positioning error accumulating over time in integrated navigation computation of SINS/VEL limits the gravimetry test within short duration. Although the gravity disturbance error caused by position error does not have a notable effect on the total statistical and relative accuracy assessments, it still cannot be neglected especially the height error for single point gravity determination [3]. For example, only 3 m height error will lead to about 1 mGal free-air reduction error which is unacceptable in geodesy applications. Facing no GNSS environments like in the test, adding altimeter sensors and landmark correction method should be considered to improve the position accuracy for further applications.

To a certain extent, the comparable agreement level between traditional SINS/GNSS method and this new SINS/VEL method suggests an important potential of land vehicle gravimetry in different conditions and environments. This method of SINS/VEL has provided a new option dealing with gravimetry in some special applications. Not only applied in land vehicle gravimetry test, this method can also be applied for underwater gravimetry, such as submarine gravity survey, which cannot get the support of GNSS either but can get Doppler velocity information. Conditions of underwater gravimetry are similar to the land vehicle environment. This method for land vehicle gravimetry still has a big potential if navigation positioning accuracy could be improved and errors could be well estimated. For the further research directions, providing accurate position should be further focused on and the low-pass filtering technology which is more suitable for land vehicle gravimetry should be noted. Considering the complicated conditions and different characteristics of different aiding sensors, maybe combining GNSS and velometer with SINS (SINS/GNSS/VEL) for land vehicle gravimetry to adapt all test conditions will show bright prospects in the near future. 

## 5. Conclusions

Based on the former research on land vehicle SINS/GNSS gravimetry test, applying GNSS for test is challenged by complicated environments. Dealing with no GNSS conditions in land vehicle gravimetry, velometer sensor was introduced to aid the strapdown gravimeter SGA-WZ02 in this paper. Theoretical analysis and practical test indicated that it was feasible for gravity determination by ground dynamic vehicle. A new method using SINS/VEL was put forward and got the preliminary results. Results showed that the internal consistency was at the level of 1.17 mGal and 1.91 mGal for the external accuracy. Comparing with the former reference profiles which were obtained by a SINS/GNSS approach, the new method showed comparable internal and external consistency accuracy. However, errors still exist in certain aspects such as positioning errors and IMU drifts errors that should be further carefully analyzed and eliminated. This new method expands the restrictive survey conditions and suggests a big potential carrying out land vehicle gravimetry test under different conditions and environments. Hopefully in the near future, more attentions will be paid to SINS/GNSS/VEL combining computation and data fusion to improve the gravimeter accuracy in many geophysical and geodesy applications.

## Figures and Tables

**Table 1:** Accuracy requirements for Equation (6).

Items	Requirement
δα,δβ,δγ	$\leq 3\times 10^{-4}(\approx 60\prime \prime )$
δθ,δψ	$\leq 1\times 10^{-3}(\approx 0.06^{\circ })$
$\delta k_{velo}$	$\leq 1\times 10^{-3}$

**Table 2:** Performance specifications of S-350 sensor.

Items	Unit	Value
Speed range	km/h	0–250
Distance resolution	mm	2.47
Measurement accuracy	%FSO (Full Scale Output)	<±0.2
Max measurement frequency	Hz	250
Working distance and range	mm	350 ± 100
Power supply	V	10–28

**Table 3:** Statistics of differences for internal and external consistency (Units: mGal).

Items		Max	Min	Mean	RMS	Total RMS
Internal	L1	2.22	−2.51	0.09	1.04	1.17
	L2	2.11	−2.55	−0.45	0.91	
	L3	3.59	−4.76	−0.27	1.38	
	L4	4.21	−2.06	0.63	1.28	
External	L1	4.69	−3.90	0.18	1.55	1.91
	L2	3.28	−5.85	−0.36	1.61	
	L3	7.54	−7.16	−0.17	2.37	
	L4	5.55	−4.32	0.72	1.98	
